# Supplementary material for: Functional reconstitution of a bacterial CO2 concentrating mechanism in Escherichia coli
Source: eLife. 2020 Oct 21;9:e59882. doi: 10.7554/eLife.59882 (PMC7714395; doi:10.7554/eLife.59882)
Supplement: Transparent reporting form [file elife-59882-transrepform.pdf]

eLIFE
Westbrook Centre, Milton Road
Cambridge CB4 1YG
UK
P 01223 855340
W elifesciences.org
T @elife

eLife's transparent reporting form

We encourage authors to provide detailed information *within their submission* to facilitate the interpretation and replication of experiments. Authors can upload supporting documentation to indicate the use of appropriate reporting guidelines for health-related research (see [EQUATOR Network]), life science research (see the [BioSharing Information Resource]), or the [ARRIVE guidelines] for reporting work involving animal research. Where applicable, authors should refer to any relevant reporting standards documents in this form.

If you have any questions, please consult our Journal Policies and/or contact us: editorial@elifesciences.org.

Sample-size estimation

- You should state whether an appropriate sample size was computed when the study was being designed
- You should state the statistical method of sample size computation and any required assumptions
- If no explicit power analysis was used, you should describe how you decided what sample (replicate) size (number) to use

Please outline where this information can be found within the submission (e.g., sections or figure legends), or explain why this information doesn't apply to your submission:

Reported data derive from microbial growth experiments. The number of replicates was chosen so that a fully controlled experiment could be set up in single workday. In Figure 4 – figure supplement 1, for example, an experiment testing 14 strains in biological quadruplicate and in two conditions required 2 x 14 x 4 = 112 precultures and 112 experimental tubes, which was near the limit of what we could manage in a single workday while maintaining sterility. Other experiments (e.g. anaerobic growth tests reported in Figure 2D and S4B) were similarly constrained by time and sample management.

Replicates

- You should report how often each experiment was performed
- You should include a definition of biological versus technical replication
- The data obtained should be provided and sufficient information should be provided to indicate the number of independent biological and/or technical replicates
- If you encountered any outliers, you should describe how these were handled
- Criteria for exclusion/inclusion of data should be clearly stated
- High-throughput sequence data should be uploaded before submission, with a private link for reviewers provided (these are available from both GEO and ArrayExpress)

Please outline where this information can be found within the submission (e.g., sections or figure legends), or explain why this information doesn't apply to your submission:

The difference between biological and technical replication is discussed in appropriate figure captions and the Methods section. The number of replicates is given in appropriate figure captions as well. These include main text Figures 2-6 and associated figure supplements . No data were excluded and source data and code is provided in the linked github repository.

Statistical reporting

- Statistical analysis methods should be described and justified
- Raw data should be presented in figures whenever informative to do so (typically when N per group is less than 10)
- For each experiment, you should identify the statistical tests used, exact values of N, definitions of center, methods of multiple test correction, and dispersion and precision measures (e.g., mean, median, SD, SEM, confidence intervals; and, for the major substantive results, a measure of effect size (e.g., Pearson's r, Cohen's d)
- Report exact p-values wherever possible alongside the summary statistics and 95% confidence intervals. These should be reported for all key questions and not only when the p-value is less than 0.05.

Please outline where this information can be found within the submission (e.g., sections or figure legends), or explain why this information doesn't apply to your submission:

Many of the data reported are binary in nature, e.g. growth vs no growth in Figure 2C-D and Figure 4B. We do not generally calculate P-values for binary results, and instead give data for proper controls with biological replication. Experimental results were also verified by conducting them in multiple modalities (full replication). For example, the CCMB1 strain was evaluated by streaking, spotting, and plate reader growth conditions (Figure 2C-D and supplements). Similarly, growth in ambient air was tested in a bioreactor, plate reader, and shaking incubator (Figures 3-4 and supplements) all of which gave qualitatively consistent results from controlled experiments. Where P-values are calculated, e.g. Figure 4 – supplement 1, we used nonparametric tests (Mann-Whitney). For the CCM genetics experiments in Figure 4, we calculated P-values using a nonparametric test (Mann-Whitney). These values are not corrected for multiple hypothesis testing because each P-value corresponds to a single hypothesis (ambient vs high CO2) that was chosen prior to the experiment. Nonetheless, this experiment was repeated over a longer time scale to ensure replicability of growth phenotypes (Figure 4 – supplement 1, panel C). When quantitative comparisons are reported, we avoided reporting P-values and instead calculated 99% confidence intervals for effect sizes by bootstrapping or sampling approaches described in the Methods section and appropriate figure legends: Figure 3 (inset), Figure 6C, and associated figure supplements. Confidence intervals are preferable to P-values as P-values do not report on the effect size and are not stable when effect sizes and/or sample sizes are small.

(For large datasets, or papers with a very large number of statistical tests, you may upload a single table file with tests, Ns, etc., with reference to sections in the manuscript.)

Group allocation

- Indicate how samples were allocated into experimental groups (in the case of clinical studies, please specify allocation to treatment method); if randomization was used, please also state if restricted randomization was applied
- Indicate if masking was used during group allocation, data collection and/or data analysis

Please outline where this information can be found within the submission (e.g., sections or figure legends), or explain why this information doesn't apply to your submission:

Since we report only reductionist experiments with laboratory strains of E. coli, we grouped samples by their genotypes. Grouping is described in figure legends and main text of the paper.

Additional data files ("source data")

- We encourage you to upload relevant additional data files, such as numerical data that are represented as a graph in a figure, or as a summary table
- Where provided, these should be in the most useful format, and they can be uploaded as "Source data" files linked to a main figure or table
- Include model definition files including the full list of parameters used
- Include code used for data analysis (e.g., R, MatLab)
- Avoid stating that data files are "available upon request"

Please indicate the figures or tables for which source data files have been provided:

All source code and data is available in a public github repository linked in the manuscript: https://github.com/flamholz/carboxecoli
